# Supplementary material for: The urinary bladder wall is remodeled by undulatory resistance training in female Wistar rats
Source: PeerJ. 2025 Mar 31;13:e19172. doi: 10.7717/peerj.19172 (PMC11967418; doi:10.7717/peerj.19172)
Supplement: Supplemental Information 3 [file peerj-13-19172-s003.pdf]

URT group	HE staining		uro=urothelium		LP= lamina propria		SM= smooth muscle layer					
	uro1	uro2	uro3	uro4	LP1	LP2	LP3	LP4	SM1	SM2	SM3	SM4
HE#1 field A section 1	150	325	301.04	550.11	2725.46	1525.2	2248.47	2825.11	4825.26	7800.16	4229.44	2350.13
HE#1 field A section 2	525.59	340.04	350.89	150	2900.43	4770.55	2925.11	1925.16	2500.12	3275.67	3800.74	4600.27
HE#1 field A section 3	520.22	201.56	175	141.42	4354.67	6126.28	7453.4	3996.56	6802.07	2200.57	1375.23	1380.67
HE#1 field A section 4	375.83	100	160.08	413.82	7250.04	3850.05	1949.68	5217.52	4325.07	5025.06	8328.45	2283.77
HE#1 field B section 1	125	176.78	176.78	276.13	3800.33	3075.41	1126.11	1950.16	5450.5	2825.11	2250.56	2925.43
HE#1 field B section 2	604.67	875	340	235.85	2508.61	5915.29	4669.92	4575.07	8819.3	8472.38	7679.72	7364.95
HE#1 field B section 3	235.85	265.75	195.26	575.54	6124.74	2759.76	3342.15	6500.43	5047.09	8516.34	6727.28	4650.07
HE#1 field B section 4	575.74	425.73	160.08	223.61	2675.12	4250.3	2770.04	2664.23	7950.16	7550.37	9419.16	7603
HE#1 field C section 1	176.78	403.11	575.74	301.04	2566.25	1435.71	1875.67	1675	3382.03	6490.62	4875.06	5400.06
HE#1 field C section 2	450.69	276.13	276.13	325.96	4075.69	2700.46	1375.91	2500.12	3926.27	4125.3	6000.47	5850.48
HE#1 field C section 3	391.31	625	425.73	520.22	1546.16	1425.22	1468.2	2047.1	9756.09	7611.59	5428.46	2148.98
HE#1 field C section 4	301.04	237.17	514.78	270.42	5075.95	3811.58	3812.56	2711.66	5275.95	6920.26	2919.34	16865.9
HE#1 field D section 1	424.26	206.16	400.78	226.38	1101.14	1225.19	2213.17	3045.18	3367.86	4125.08	6063.47	3398.62
HE#1 field D section 2	175	388.1	498.12	416.08	2557.95	3293.36	4114.91	3792.92	8909.62	9327.51	7894.46	6598.72
HE#1 field D section 3	285.04	176.78	475.66	375.83	4452.6	3572.46	5680.5	4627.43	3716.52	8752.61	2650.47	6180.06
HE#1 field D section 4	371.65	452.77	350.89	437.32	3469.33	2630.83	3476.44	2593.86	6177.78	6045.71	8230.47	6950.04
HE#2 field A section 1	675.46	360.56	485.41	318.2	776.61	1712.64	1505.41	8178.52	7675.16	8216.79	8771.3	9396.71
HE#2 field A section 2	502.49	512.96	751.66	650.48	3622.15	2315.84	3325.38	850.37	7095.82	5303.77	6100.05	7825.04
HE#2 field A section 3	1015.2	785.02	400.78	400.78	1995.31	3420.62	4876.03	1325.24	6040.07	6067.38	9825.29	9725.29
HE#2 field A section 4	525.59	425.73	301.04	689.66	2003.9	1500.21	1935.36	1540.5	4492.84	5225.24	4310.23	5773.76
HE#2 field B section 1	400.78	503.12	251.25	375.83	3150.4	1241.47	1850.68	1950.16	4475.63	4641.19	8802.27	6827.24
HE#2 field B section 2	458.94	328.82	251.25	276.13	1330.88	1525	3251.54	2400.52	6946.22	5900.69	7427.06	3750.08
HE#2 field B section 3	375.83	301.04	355.32	395.28	2275	3204.78	1523.98	1858.09	3725.08	6138.05	4751.64	4238.07
HE#2 field B section 4	506.21	525.59	276.13	700.45	1694.48	1177.39	4154.82	1625.77	4843.62	5753.48	5176.51	6225.05
HE#2 field C section 1	450.69	794.51	500	656.22	2250.14	1858.26	3545.51	3660	7550.66	8059.35	8332.84	9950.94
HE#2 field C section 2	686.48	903.12	403.11	475.66	905.54	1364.05	4225.67	3575.35	7623.65	6174.65	7901.42	3500.8
HE#2 field C section 3	400.78	1050	650.48	875.36	875.36	4457.02	4200.07	1725.72	8951.26	6253.2	6800.41	5450.52
HE#2 field C section 4	693.27	180.28	665.68	371.65	1100.28	2380.26	1755.35	4205.43	4707.71	3991.63	5500	4634.99
HE#2 field D section 1	301.04	279.51	340.04	502.49	5051.55	1672.2	3544.01	1275.98	4502.5	4902.87	8318.65	8778.56
HE#2 field D section 2	357.95	335.41	325.96	477.62	1234.91	2090.75	3352.33	1825.68	6302.88	6367.94	7350.04	4201.86
HE#2 field D section 3	575.54	803.51	500	700.45	2250.56	1350.93	1855.06	3200.88	4575.63	5981.33	4330.49	4001.25
HE#2 field D section 4	502.49	206.16	301.04	604.15	1623.46	4766.16	4229.73	1355.77	4850.06	5845.08	5204.87	6572.15

HE#3 field A section 1	700.45	520.22	552.27	425.73	2027.47	4654.9	2925.43	704.01	5876.91	7063.86	4079.91	4529.42
HE#3 field A section 2	279.51	215.06	388.1	400.78	1690.6	535.02	43	1250.25	2655.42	2706.13	5012.61	5726.96
HE#3 field A section 3	301.04	301.04	357.95	400.78	525.59	1475.85	1484.08	400.78	4754.21	2625.12	2670.79	2601.08
HE#3 field A section 4	336.34	406.97	347.31	390.51	2483.07	3237.38	1761.39	2408.32	4279.75	4525.62	4473.32	3945.01
HE#3 field B section 1	425.73	427.93	350.89	548.29	2502	2950.11	3428.28	3099.7	3850.73	3851.3	5700.22	3272.71
HE#3 field B section 2	230.49	427.93	437.32	832.54	4857.02	3075.1	2961.52	3847.81	3140.16	3250.38	6809	6115.25
HE#3 field B section 3	347.31	381.61	336.34	400	1657.18	1325.94	3129	1125.28	9724.71	8905.41	3921.18	3078.66
HE#3 field B section 4	201.56	650.48	838.53	460.98	2600.12	4930.14	3298.67	3735.05	6125.46	5450.23	3178.54	1882.15
HE#3 field C section 1	400.78	475	450.69	125	1977.53	5942.06	500.62	400.78	4450.07	3350.84	4429.52	7654.94
HE#3 field C section 2	276.13	251.25	250	226.38	1250.25	1378.63	500.62	1825.68	2253.47	2953.81	2406.37	3728.02
HE#3 field C section 3	201.56	301.04	400.78	325.96	700.45	2502	3200.88	2100	2326.21	3552.2	5653.54	6475.05
HE#3 field C section 4	328.82	350.89	550.57	279.51	2279.94	2652.95	3250.1	2639.72	6139.73	4701.66	2551.1	2450.13
HE#3 field D section 1	585.23	673.15	419.08	679.15	2531.06	1667.71	2151.31	1856.07	4665.63	4354.88	3409.18	3407.44
HE#3 field D section 2	803.51	878.21	355.32	498.12	3448.28	3225.39	2245.69	1534.8	2918.15	8550.04	5097.18	2857.12
HE#3 field D section 3	492.44	388.91	412.31	1450.86	4296.07	2970.69	2270.06	2175.14	5047.77	3307	2985.17	5577.02
HE#3 field D section 4	456.21	285.04	825.38	693.27	2694.67	4917.44	3348.97	2063.52	7645.14	1391.49	2479.04	5850.75
HE#4 field A section 1	176.78	200	400.78	525.59	1151.09	5436.05	1701.65	1901.48	12400.03	749.56	7734.1	6877.23
HE#4 field A section 2	230.49	660.02	254.95	304.14	919.24	1375.91	2144.91	1665.27	11267.49	10107.42	10307.76	6802.25
HE#4 field A section 3	395.28	416.08	206.16	201.56	2650.47	1957.36	3304.64	4589.39	6483.3	3132.49	6142.58	7152.49
HE#4 field A section 4	450.69	226.38	328.82	201.56	1325.24	3975.08	1775.18	5125.55	4750.07	5152.97	2225.56	5101.53
HE#4 field B section 1	502.49	304.14	276.13	452.77	1826.54	875.36	1528.28	1559.85	7654.08	6225	3735.14	5452.81
HE#4 field B section 2	180.28	388.91	450.69	620.99	2850.99	3485.77	4319.87	2967.43	7980.76	9749.1	8163.68	7780.1
HE#4 field B section 3	251.25	400.78	279.51	825.38	2766.32	3175.89	5620.05	2704.16	5246.9	5425.92	3300.09	5981.27
HE#4 field B section 4	485.41	605.19	355.32	336.34	2042.21	1987.78	2674.07	5827.79	6133.77	8892.31	8036.05	4920.62
HE#4 field C section 1	400.78	190.39	304.14	223.61	800.39	2363.52	1578.77	2285.01	12036.46	10611.7	7585.39	4640.04
HE#4 field C section 2	301.04	665.68	279.51	190.39	1133.58	1440.7	3349.07	1837.46	11256.25	10707.15	11169.85	8547.51
HE#4 field C section 3	548.29	355.32	325.96	276.13	2547.79	2580.82	1159.74	4630.47	6124.74	4854.12	5782.79	6072.74
HE#4 field C section 4	276.13	325.96	251.25	375.83	1476.91	1478.39	4362.12	2278.43	5425.06	4926.02	4583.26	6954.49
HE#4 field D section 1	701.78	539.68	416.08	592.66	725.43	656.22	380.79	2001.41	7181.27	5381.1	3553.17	4706.98
HE#4 field D section 2	270.42	381.61	520.22	458.94	3654.62	3978.85	4227.44	2985.9	7637.49	8497.79	7808.21	9430.6
HE#4 field D section 3	500.62	525.59	625.5	480.23	3027.58	2875.98	4750.07	2935.34	5503.64	5580.6	3825.08	6094.77
HE#4 field D section 4	550.57	247.49	388.91	424.26	3002.6	2295.78	2687.24	6063.47	5868.24	5227.93	8293.11	4985.1

HE#5 field A section 1	350.89	279.51	347.31	127.48	1900.16	4269.15	5218.78	2050.61	6300.45	4511.17	4458.49	5200.06
HE#5 field A section 2	213.6	291.55	176.78	226.38	2454.59	3115.79	2525.5	5497.78	4480.03	2580.82	4775.59	3103.63
HE#5 field A section 3	477.62	425.73	285.41	251.25	3600.09	3400.83	1806.24	3500.09	4300.07	2725.11	3483.62	5200.54
HE#5 field A section 4	176.78	251.25	340.04	152.07	3528.19	3700.34	3835.12	2814.47	5902.59	7077.16	4200.07	5455.5
HE#5 field B section 1	251.25	291.55	325.96	627	2325.54	2971.74	1750.18	2977.62	2625.48	3575.26	4801.04	8102.47
HE#5 field B section 2	247.49	206.16	325.96	261.01	5155.16	4124.39	2800.45	2521.04	3248.94	2397	2150.58	2934.71
HE#5 field B section 3	475.66	257.39	371.65	336.34	5185.2	4899.23	5675.22	5396.12	5807.75	6808.13	3207.9	3285
HE#5 field B section 4	176.78	391.31	279.51	515.39	1151.09	1756.59	3037.47	2023.92	4450.28	3488.28	3945.88	4181.58
HE#5 field C section 1	276.13	223.61	301.04	447.21	1350.23	5006.06	2850.11	1398.66	5350.93	4290.47	3576.4	3893.91
HE#5 field C section 2	309.23	145.77	257.39	301.04	2288.7	1285.74	4190.76	2450.13	4952.9	4880.83	5415.08	5600.06
HE#5 field C section 3	480.88	302.08	265.75	176.78	1644.12	3844.23	1820.89	1202.34	4928.62	3453.98	3538.36	4825.26
HE#5 field C section 4	328.82	201.56	226.38	176.78	2510.1	4178.67	3575.79	3501.43	5393.8	5526.41	3800.33	5725.22
HE#5 field D section 1	375.83	176.78	625.5	525.59	2730.61	3058.29	2900.11	2875.11	2956.77	4021.58	7957.7	8625.33
HE#5 field D section 2	237.17	230.49	279.51	276.13	4572.81	3961.14	3349.72	1575.2	3635.93	2886.17	4555.56	3825.33
HE#5 field D section 3	575.54	335.41	442.3	201.56	5136.94	5305.19	4374.43	3475.36	5917.14	6989.46	4236.3	3325.09
HE#5 field D section 4	300	725.43	425.73	450.69	925.34	2178.59	2950.95	1727.9	4825.06	3575.35	4727.38	4401.78
HE#6 field A section 1	350.89	445.11	375.83	427.93	2851.75	1065.95	2025.15	2325.54	2576.09	4911.47	4301.16	3627.15
HE#6 field A section 2	223.61	257.39	103.08	261.01	1160.01	2015.56	1951.44	1820.03	3450.09	3975	6702.29	4187.56
HE#6 field A section 3	145.77	305.16	190.39	350.89	2436.31	3200.88	2246.11	1600.2	3537.39	2885.85	6578.47	6375.2
HE#6 field A section 4	375.83	348.21	257.39	450.69	675.46	2360.75	3210.33	1825.17	6400.78	4993.06	5011.99	3777.07
HE#6 field B section 1	425.73	378.32	325.96	301.04	2025.62	1981.16	4800.59	2701.04	3400.37	3716.52	8051.9	5376.45
HE#6 field B section 2	301.04	375	176.78	276.13	2000	1200	1801.56	625	3150.1	3450.36	2650.47	3977.83
HE#6 field B section 3	226.38	225	201.56	152.07	2150.15	977.88	201.56	1200	3275.1	4031.28	3470.32	3150.1
HE#6 field B section 4	279.51	176.78	201.56	301.04	550.57	850.37	1000.31	1646.4	4450.07	4050.08	3850.11	2090.75
HE#6 field C section 1	452.77	378.32	261.01	182	1850.17	2043.59	2790.39	1202.6	4552.47	5284.17	3198.63	3602.52
HE#6 field C section 2	265.75	395.28	427.93	226.38	1101.14	991.53	1127.5	2126.32	3606.68	4238.9	6536.28	3407.44
HE#6 field C section 3	382.43	237.17	442.3	625.5	4268.78	2363.52	1063.01	975.32	2301.22	6266.68	7586.58	6575.05
HE#6 field C section 4	388.1	269.26	395.28	520.22	3655.99	1931.32	2748.29	3205.56	5456.93	6662.68	5078.94	6085.07
HE#6 field D section 1	400.78	400	279.51	375	3827.04	1850.68	2075.76	2350.53	8779.31	3875.08	3354.57	2300.14
HE#6 field D section 2	350.89	500.62	152.07	450.69	2075.15	1425.88	2730.61	850.37	3300.38	2777.81	3930.09	4125.08
HE#6 field D section 3	403.11	275	251.25	375.83	2503.12	2100.15	975.32	1000.31	2303.39	3400.37	4103.73	3050.41
HE#6 field D section 4	226.38	200	176.78	302.08	1532.36	926.35	775.4	1075.29	4144.35	3951.98	3300.09	3245.86

HE#7 field A section 1	206.16	213.6	279.51	176.78	1450.86	2719.6	5760.32	1435.71	3777.07	3895.03	1861.62	1860.78
HE#7 field A section 2	226.38	206.16	223.61	534.44	2504.5	1237.44	3957.9	1488.5	6702.98	8993.47	6026.24	5968.25
HE#7 field A section 3	355.32	357.95	566.79	608.79	2094.04	3312.57	2461.83	2110.84	4042.97	5416.87	5252.44	5518.27
HE#7 field A section 4	700.45	564.03	548.29	400.78	1912.13	1947.11	2512.47	4201.19	6381.86	4372.71	3274.24	3302.37
HE#7 field B section 1	583.63	494.97	700.45	514.78	1716.46	3934.62	3288.24	1600.2	3834.38	4158.27	3288.24	3169.09
HE#7 field B section 2	514.78	570.64	583.1	302.08	2384.59	3221.51	2873.37	3315.21	4695.74	4104.42	4970.16	5115.72
HE#7 field B section 3	425	781.02	336.34	577.17	3749.17	3836.1	4763.4	4400.64	4419.42	4138.99	5657.3	3450.82
HE#7 field B section 4	673.15	651.92	500.62	395.28	3005.2	1415.1	1333.46	2389.69	5670.21	6667.32	7365.88	4160.6
HE#7 field C section 1	125	325.96	201.56	176.78	1470.76	1666.21	1308.63	1191.9	3300.47	2855.04	3864.34	3910.4
HE#7 field C section 2	269.26	450.69	552.27	355.32	2438.24	1603.12	4214.56	3092.83	4550.62	9187.27	5511.13	8645.23
HE#7 field C section 3	403.11	335.41	452.77	596.34	1397.54	3227.42	1758.73	3259.03	5524.72	5966.26	3087.27	1610.32
HE#7 field C section 4	313.25	445.11	536.77	328.82	3829.57	2015.56	2353.85	2910.76	7468.77	5643.86	3305.77	3029.95
HE#7 field D section 1	602.08	494.97	749.17	682.83	2801.79	3623.53	1991.23	2505.62	5396.82	3506.16	2885.31	2830.42
HE#7 field D section 2	190.39	254.95	391.31	506.21	3590	2478.15	3105.84	1442.44	3927.87	7331.14	4833.22	5371.22
HE#7 field D section 3	620.99	540.83	772.17	378.32	3768.7	4716.79	2787.58	2951.69	4119.62	5145.02	6067.38	4531.9
HE#7 field D section 4	525.59	590.02	375.83	585.77	1877.66	2234.39	2600.12	1567.04	6675.42	3155.25	3500.09	2683.4

SEDENTARY group (control)	uro 1	uro 2	uro 3	uro 4	LP 1	LP 2	LP 3	LP 4	SM1	SM2	SM3	SM4
HE#1 field A section 1	276.13	225	103.08	375.83	2826	6125.05	4701.06	4002.81	3525.35	6752.27	7625.66	8850.56
HE#1 field A section 2	26.38	201.56	152.07	201.56	1980.69	1950.16	1550.2	1425.88	3076.63	3075.91	3876.29	4275.29
HE#1 field A section 3	250	269.26	292.62	503.12	2721.33	7614.21	8001.56	3092.83	9714.74	10654.96	12687.64	9013.88
HE#1 field A section 4	182	206.16	279.51	305.16	1019.8	1401.12	1566.25	1945.83	4512.76	4038.87	2996.66	2245.69
HE#1 field B section 1	353.55	158.11	201.56	246.22	2725.46	5926.9	9025.87	3000.42	9800.29	9233.67	6875.18	3530.67
HE#1 field B section 2	412.31	213.6	235.85	90.14	2366.7	4754.6	3784.51	3644.34	4900.26	4956.12	4209.29	2848.79
HE#1 field B section 3	246.22	492.44	261.01	279.51	1097.72	7236.8	6842.88	2838.68	9298.82	11887.02	13015.78	9295.64
HE#1 field B section 4	201.56	176.78	127.48	246.22	1831.15	1219.12	1861.62	1369.53	3594.61	3795.56	3457.33	3862.64
HE#1 field C section 1	251.25	206.16	304.14	226.38	1779.22	4625.07	3125.1	304.14	10176.93	9900.03	9800.51	5491.47
HE#1 field C section 2	160.08	212.13	301.04	328.82	2687.01	2665.17	1438.97	1525.82	4833.74	3156.14	3304.92	3650.09
HE#1 field C section 3	246.22	382.43	403.11	357.95	7468.77	6846.35	4769.76	1867.65	11314.7	12700.1	10463.57	8913.09
HE#1 field C section 4	301.04	152.07	176.78	127.48	1475.21	1781.33	1201.04	1451.94	3225.1	4119.47	4432.06	4339.14
HE#1 field D section 1	328.82	251.25	226.38	275	2431.31	4851.03	7630.9	3076.63	8975.03	5575.9	3950	3500.8
HE#1 field D section 2	336.34	348.21	212.13	176.78	1660.01	2360.08	3226.84	1525.82	5186.52	4865.31	5485.66	3307.66
HE#1 field D section 3	38891	320.16	265.75	215.06	7572.48	7167.03	1567.04	2809.03	12211.47	13027.02	10400.96	9147.57
HE#1 field D section 4	190.39	270.42	111.8	190.39	982.34	1612.45	1331.35	1574.21	3973.98	3786.24	4049.46	3985.29
HE#2 field A section 1	251.25	250	276.13	348.21	400	11352.59	6109.67	4384.63	5275.53	6803.31	7107.13	5650.5
HE#2 field A section 2	427.2	279.61	167.71	235.85	4690.68	5580.88	5673.02	4911.72	7031.45	5671.92	4813	6806.11
HE#2 field A section 3	340.04	371.65	328.82	182	6791.54	2406.5	1052.68	5152.18	9253.78	6123.37	5925.21	8775.32
HE#2 field A section 4	292.62	261.01	237.17	213.6	2725.86	1156.77	1091.44	3129	7311.98	8941.93	9946.36	4527.49
HE#2 field B section 1	201.56	265.75	353.55	226.38	4912.29	4835.61	4565.43	3428.28	8308.17	6832.73	5749.13	5484.64
HE#2 field B section 2	301.04	279.51	395.28	195.26	4056.25	5008.49	3198.63	4981.97	5701.37	7078.22	5724.13	6459.34
HE#2 field B section 3	425.73	450.69	285.04	375.83	4007.8	2860.94	1068	800.39	5103.92	8229.6	7874.64	6325.05
HE#2 field B section 4	333.54	364.01	230.49	340.04	3067.27	6785.37	1060.66	4447.26	4376.79	3676.36	9212.25	5651.38
HE#2 field C section 1	261.01	201.56	375.83	427.93	1085.41	2128.67	3811.82	1403.57	8821.32	7490.2	7247.11	6041.78
HE#2 field C section 2	292.62	570.09	213.6	304.14	1420.61	1434.62	2270.05	3637.39	7647.96	7162.1	5165.09	5535.55
HE#2 field C section 3	320.16	340.04	627	237.17	3962.95	6033.09	1525.2	3723.24	5568.21	6324.56	4377.57	4337.22
HE#2 field C section 4	360.56	160.08	285.04	235.85	3810.84	6257.99	3843.99	2823.12	6035	7566.13	4485.67	3345.52
HE#2 field D section 1	302.08	215.06	350.89	552.27	1930.84	1803.47	2276.24	1425.22	6230.67	7930.95	7950.04	6200.81
HE#2 field D section 2	251.25	336.34	226.38	456.21	2378.29	749.17	3006.66	3275.1	4877.31	6815.12	6152.49	9600.13
HE#2 field D section 3	540.83	424.26	485.41	776.61	2819.24	2227.39	4639.64	1827.74	5803.55	5922.68	6724.02	4059.33
HE#2 field D section 4	176.78	304.14	254.95	279.51	3675.34	3302.37	4434.66	3792.51	6075.46	8986.27	2733.24	5518.27

HE#3 field A section 1	400.78	176.78	182	125	2201.28	2150.58	4784.41	6018.77	4127.73	4303.56	3941.61	4002.81
HE#3 field A section 2	158.11	152.07	226.38	279.51	3250.32	1929.05	928.04	1400.22	4428.46	5228.83	4253.6	5005.06
HE#3 field A section 3	375	103.08	503.12	251.25	2256.8	2007.02	3387.66	2786.57	1730.07	5111.08	776.61	2098.81
HE#3 field A section 4	270.42	335.41	127.48	381.61	6039.71	7105.02	6508.94	2267.43	2739.75	3834.47	4210.85	4322.18
HE#3 field B section 1	305.16	282.84	176.78	175	3319.36	5896.66	3650.09	2375.13	6048.35	3790.12	6025.83	5201.5
HE#3 field B section 2	201.56	176.78	309.23	276.13	925.34	3603.12	1950.64	1225.26	6575	4076.92	2275	3575.79
HE#3 field B section 3	160.08	320.16	353.55	176.78	1187.7	1155.69	3022	4206.02	2627.97	3945.02	1078.48	1925.65
HE#3 field B section 4	325.96	650.48	388.1	382.43	3600.09	4325.65	6763.37	6704.1	4076.92	4200.3	2603	5944.38
HE#3 field C section 1	201.56	213.6	279.51	226.38	5858.54	4423.02	3875.32	2550.12	3835.12	4118.93	4001.95	4400.28
HE#3 field C section 2	127.48	276.13	575.54	375.83	2675.47	2550.12	1775.7	1425.88	4954.04	4028.8	2526.98	4200.07
HE#3 field C section 3	201.56	285.04	305.16	357.95	2988	5403.7	3612.91	2100.3	1732.77	919.92	1026.22	3050
HE#3 field C section 4	325	536.77	305.16	485.41	3490.43	6954.58	5955.3	2343.61	4354.6	3896.79	4215.74	4632.49
HE#3 field D section 1	269.26	335.41	176.78	301.04	3830.31	5000.81	3671.51	2302.17	4806.9	3962.4	4808.85	4626.69
HE#3 field D section 2	176.78	226.38	127.48	201.56	1300.96	2400.52	2775.11	1350.23	6476.74	2300.14	2401.17	4829.14
HE#3 field D section 3	215.06	223.61	458.94	226.38	1905.42	2527.97	3366.19	3225.1	2776.8	1282.82	1621.15	1475.21
HE#3 field D section 4	500.62	257.39	302.08	529.74	5293.21	2739.75	3980.26	3148.11	1771.48	6478.09	6710.3	5859.02
HE#4 field A section 1	350.89	328.82	375.83	279.51	1026.22	5785.6	4875.58	5125.55	254.25	3480.75	1950.64	2825.44
HE#4 field A section 2	602.08	375.83	369.12	125	2026.39	2955.19	2237.47	2267.16	3895.59	3666.74	4130.98	6255.85
HE#4 field A section 3	103.08	213.6	302.08	176.78	2650	2596.75	1688.38	3605.03	3275.67	3868.54	5132.37	3932.64
HE#4 field A section 4	427.93	475.66	375.83	230.49	3576.4	3300.85	5200.96	2950.42	3276.53	4230.99	5506.87	6325.2
HE#4 field B section 1	195.26	625.5	550.57	485.41	743.3	1700.74	1625.19	4358.68	617.45	3900.08	5677.7	2383.54
HE#4 field B section 2	279.51	226.38	375.83	700	3677.13	6510.81	1751.61	1400.22	7300.17	4957.63	7051.6	7350.17
HE#4 field B section 3	246.22	509.9	180.28	237.17	850.37	735.7	1320.04	2189.89	877.85	2433.11	3613.34	4159.48
HE#4 field B section 4	254.95	206.16	348.21	261.01	2253.47	2159.43	2452.04	951.97	4426.98	4436.29	4069.55	5982.06
HE#4 field C section 1	223.61	226.38	350.89	500.62	2107.72	2626.9	1600.78	4250.66	1416.2	4025.7	4181.06	2300.14
HE#4 field C section 2	575.54	753.74	160.08	292.62	1500.21	1904.11	2061.55	3010.4	7304.28	5279.8	4126.89	4911.47
HE#4 field C section 3	167.71	195.26	152.07	276.13	664.27	2079.81	2241.93	1662.82	1013.04	1209.34	4455.33	4797.72
HE#4 field C section 4	403.89	425.73	350.89	378.32	951.97	2007.02	2515.08	3411.1	4734.05	4787.03	6534.95	3616.97
HE#4 field D section 1	475.66	328.82	301.04	276.13	1650.76	1475.21	4026.94	3725.34	3877.9	3100.1	3225.87	3926.27
HE#4 field D section 2	450.69	378.32	400.78	305.16	2579.37	851.47	2995.41	3811.58	3350.37	5931.38	5948.79	2820.13
HE#4 field D section 3	378.32	388.1	257.39	301.04	1015.2	735.27	3141.76	2183.03	883.88	3005.2	2551.1	4330.49
HE#4 field D section 4	226.38	301.04	575.54	450.69	1625.77	2654.24	4575.07	3103.63	6291.11	4901.59	6650.42	4504.44

HE#5 field A section 1	257.39	291.55	425	347.31	1325	3198.63	704.45	2165.21	7225.39	6275.05	5292.09	3848.54
HE#5 field A section 2	427.2	190.39	412.31	425	2655.77	515.39	538.52	2282.54	2429.25	1922.4	2958.25	8741.64
HE#5 field A section 3	301.04	333.54	436.61	482.83	3096.07	1431.13	862.05	1231.11	3701.77	6487.01	6813.68	6627.83
HE#5 field A section 4	70.71	55.9	378.32	291.55	3275.1	4075.08	1814.01	2683.86	2550.12	2476.14	3067.27	2617.37
HE#5 field B section 1	357.95	212.13	390.51	475.66	1971.36	515.39	2176.15	1575.79	5114.99	7448.91	6558.44	6150.81
HE#5 field B section 2	425.73	388.1	103.08	201.56	651.92	2516.57	3131.29	3972.56	6874.09	3642.89	2107.28	7359.73
HE#5 field B section 3	492.44	493.08	223.61	469.71	2340.94	1346.29	1923.05	6046.69	3830.88	6329	5076.54	5557.71
HE#5 field B section 4	254.95	50	498.12	127.48	4620.88	4451.47	2716.73	3137.87	3783.6	2741.46	2548.04	2221.06
HE#5 field C section 1	375.83	340.04	270.42	535.02	2880.32	1578.17	471.7	2135.71	6534.38	6917.05	7194.88	6510.81
HE#5 field C section 2	369.12	195.26	625	246.22	2700.12	3322.74	2121.91	3085.85	1625	6200.05	4103.12	2508.61
HE#5 field C section 3	265.75	570.09	261.01	1049.4	3448.28	2335.19	2159.43	1861.62	3785.66	4026.86	6476.93	6933.7
HE#5 field C section 4	276.13	201.56	502.49	427.2	4026.94	2175.57	2080.41	2590.49	4875.58	2427.06	3062.37	2561.49
HE#5 field D section 1	395.28	375	412.31	425.73	1947.43	1581.14	1948.24	2326.48	7302.61	7025.4	5418.08	6703.82
HE#5 field D section 2	79.06	160.08	291.55	223.61	3578.06	3660	1595.31	2589.64	1608.77	6030.6	4099.39	3042.72
HE#5 field D section 3	282.84	336.34	443	378.32	5108.82	3554.31	1138.53	3766.3	3666.23	3859.57	6798.39	5764.66
HE#5 field D section 4	226.38	55.9	350.89	355.32	4407.73	3407.8	1754.46	2289.65	4772.97	3020.76	3407.44	2616.41

outlier
