# Supplementary material for: The urinary bladder wall is remodeled by undulatory resistance training in female Wistar rats
Source: PeerJ. 2025 Mar 31;13:e19172. doi: 10.7717/peerj.19172 (PMC11967418; doi:10.7717/peerj.19172)
Supplement: Supplemental Information 4 [file peerj-13-19172-s004.pdf]

URT group					SEDENTARY group (control)				
Rat#	Uro	LP	SM	Total bladder wall	Rat#	Uro	LP	SM	Total bladder wall
1	343.44	3323.47	5683.12	9350.03	1	248.38	3294.26	6448.98	9991.62
2	499.28	2493.44	6233.39	9226.11	2	303.21	3286.95	6413.80	10003.96
3	448.63	2422.00	4381.28	7251.91	3	270.11	3370.82	3783.86	7424.79
4	387.65	2636.92	6748.70	9773.27	4	318.92	2520.23	4142.62	6981.77
5	317.47	3143.19	4537.18	7997.84	5	318.66	2477.77	4707.20	7503.63
6	315.04	1875.03	4309.61	6499.68					
7	451.62	2658.05	4702.12	7811.79					
**Mean**	**394.73**	**2650.30**	**5227.91**	**8272.95**	**Mean**	**291.86**	**2990.01**	**5099.29**	**8381.15**
**SD**	**73.11**	**478.56**	**986.97**	**1210.71**	**SD**	**31.40**	**449.68**	**1259.85**	**1489.13**
**SEM**	**27.63**	**180.88**	**373.04**	**457.61**	**SEM**	**14.04**	**201.10**	**563.42**	**665.96**

Unpaired Student t-test	URT vs. SED	Uro	p=	0.009
		LP	p=	0.240
		SM	p=	0.854
		total wall	p=	0.897

%URT/SEDENTARY			
**Uro**	**LP**	**SM**	**Total wall**
**35.25**	**-11.36**	**2.52**	**-1.29**

Ratio layer/total wall						
**URT group**				**SED group**		
**Uro**	**LP**	**SM**		**Uro**	**LP**	**SM**
0.036731	0.35545	0.607818		0.024859	0.329702	0.645439
0.054116	0.270259	0.675625		0.030309	0.328565	0.641126
0.061864	0.333981	0.604155		0.036379	0.453995	0.509625
0.039664	0.269809	0.690526		0.045679	0.360973	0.593348
0.039694	0.393005	0.567301		0.042467	0.33021	0.627323
0.04847	0.28848	0.66305				
0.057813	0.340261	0.601926				
**0.048**	**0.320**	**0.632**		**0.035**	**0.357**	**0.608**
**0.010**	**0.047**	**0.046**		**0.009**	**0.054**	**0.056**
**0.004**	**0.018**	**0.017**		**0.004**	**0.024**	**0.025**

Unpaired Student t-test	URT vs. SED		Uro	p=	0.044
			LP	p=	0.228
			SM	p=	0.410
