# Supplementary material for: The urinary bladder wall is remodeled by undulatory resistance training in female Wistar rats
Source: PeerJ. 2025 Mar 31;13:e19172. doi: 10.7717/peerj.19172 (PMC11967418; doi:10.7717/peerj.19172)
Supplement: Supplemental Information 5 [file peerj-13-19172-s005.pdf]

Quantitative analysis- Image J

MASSON TRICHROME

**URT**			**SED**		
**ANIMAL**	**Pixels/um2**		**ANIMAL**	**Pixels/um2**	
R1	140,236		R2	131,374	
R2	182,573		R3	105,244	
R3	149,708		R4	126,502	
R4	141,652		R5	139,696	
R5	182,262				
R6	158,165				
R7	175,057				
R8	158,859				
**MEAN**	161.1		**MEAN**	125.7	
**SD**	17.16		**SD**	14.69	
**SE**	6.066		**SE**	7.344	

**unpaired Student t-test**		
**Masson**	**SED vs. URT**	**p=0.0056**
		**F=1.365**

Quantitative analysis- Image J

RESORCIN-FUCHSIN

**URT**		**SED**		
**ANIMAL**	**Pixels/um2**	**ANIMAL**	**Pixels/um2**	
R1	28.24	R1	19.53	
R2	21.96	R2	8.87	
R5	21.35	R4	10.73	
R6	18.97			
R7	19.09			
R8	18.88			
**MEAN**	21.42	**MEAN**	13.04	
**SD**	3.597	**SD**	5.694	
**SE**	1.468	**SE**	3.287	

**unpaired Student t-test**		
**Resorcin-Fuchsin**	**SED vs. URT**	**p=0.0284**
		**F=2.506**

Quantitative analysis- Image J

MMP1

URT | **SED**

ANIMAL	Pixels/um2	ANIMAL	Pixels/um2
R1	75,925	R1	31,258
R2	50,211	R2	13,364
R3	56,493	R3	30,600
R4	67,849	R4	1,879
R5	69,755		
R7	61,087		
R8	58,458		
**MEAN**	62.83	**MEAN**	19.28
**SD**	8.815	**SD**	14.25
**SE**	3.332	**SE**	7.126

unpaired Student t-test

**MMP1**	**SED vs. URT**	**p=0.0001**
		**F=2.615**

Quantitative analysis- Image J

TIMP1

URT

ANIMAL	Pixels/um2
R1	32,368
R3	27,712
R4	31,309
R5	49,123
R6	50,437
R8	42,474
**MEAN**	38.9
**SD**	9.755
**SE**	3.983

SED

ANIMAL	Pixels/um2
R1	27,094
R2	20,342
R3	24,265
R4	99,007
**MEAN**	42.68
**SD**	37.66
**SE**	18.83

unpaired Student t-test

**MMP1**	**SED vs. URT**	**p=0.8161**
		**F=14.90**
